# Comparative Transcriptome Analysis Reveals Differentially Expressed Genes Related to Antimicrobial Properties of Lysostaphin in *Staphylococcus aureus*

**DOI:** 10.3390/antibiotics11020125

**Published:** 2022-01-18

**Authors:** Xianghe Yan, Yanping Xie, Charles Li, David M. Donovan, Andrew Gehring, Peter Irwin, Yiping He

**Affiliations:** 1 Environmental Microbial and Food Safety Laboratory, Beltsville Agricultural Research Center, Agricultural Research Service, United States Department of Agriculture, Beltsville, MD 20705, USA; 2 Molecular Characterization of Foodborne Pathogens Research Unit, Eastern Regional Research Center, Agricultural Research Service, United States Department of Agriculture, Wyndmoor, PA 19038, USA; xieyanping@gmail.com (Y.X.); andrew.gehring@USDA.gov (A.G.); peterirwin1@comcast.net (P.I.); 3 Animal Biosciences and Biotechnology Laboratory, Beltsville Agricultural Research Center, Agricultural Research Service, United States Department of Agriculture, Beltsville, MD 20705, USA; charles.li@usda.gov (C.L.); david.donovan@usda.gov (D.M.D.)

**Keywords:** *Staphylococcus aureus*, lysostaphin, peptidoglycan hydrolase (PGH), genomics, gene expression, RNA-seq

## Abstract

Comparative transcriptome analysis and *de novo* short-read assembly of *S. aureus* Newman strains revealed significant transcriptional changes in response to the exposure to triple-acting staphylolytic peptidoglycan hydrolase (PGH) 1801. Most altered transcriptions were associated with the membrane, cell wall, and related genes, including amidase, peptidase, holin, and phospholipase D/transphosphatidylase. The differential expression of genes obtained from RNA-seq was confirmed by reverse transcription quantitative PCR. Moreover, some of these gene expression changes were consistent with the observed structural perturbations at the DNA and RNA levels. These structural changes in the genes encoding membrane/cell surface proteins and altered gene expressions are the candidates for resistance to these novel antimicrobials. The findings in this study could provide insight into the design of new antimicrobial agents.

## 1. Introduction

Lysostaphin [1], classified as a prototype III bacteriocin, is a glycyl-glycine bacteriocin peptidoglycan hydrolase (PGH) secreted by *Staphylococcus simulans*. PGH is known to degrade the peptidoglycans in *Staphylococcus aureus* cell walls, resulting in cell lysis [2]. The antimicrobial properties of lysostaphin have been demonstrated *in vitro* and *in vivo* [3]. Several mechanisms of resistance to lysostaphin have been proposed: for example, reduced *S. aureus* fitness [4]; mutations in lysostaphin-resistant *S. aureus femA* [5]; replacing the Gly3 of the pentaglycine ligand by serine in the peptidoglycan pentaglycine cross bridge or a shortened cross-bridge [6,7,8,9,10]; alterations in the plasmid-borne *lss* (lysostaphin endopeptidase) and *lif* (pACK1) genes [9,11].

In this study, we have identified the genetic mechanisms of resistance to peptidoglycan hydrolases *via* repeated exposure of *S. aureus* Newman_2010 strain (cultured wild-type (WT) *S. aureus* Newman in the year of 2010) to sublethal concentrations of a genetically engineered, triple-acting, staphylolytic, peptidoglycan hydrolase (PGH1801) [2,12]. The resultant mutant strain *S. aureus* Newman 1801_2010 has resistance to lysostaphin with a >2-fold increase in minimum inhibitory concentration (MIC) relative to the wild-type strain Newman_2010 without exposure to any PGH [2,13,14,15]. This phenotype does not appear to be caused by the resistance mechanisms described previously because no changes were found in the known resistance genes (data not shown).

Environmental conditions experienced by multiple generations of *S. aureus* Newman_2010 cultured with PGH1801 could alter this organism’s differential survival and reproduction characteristics. In response to sublethal concentrations of triple-acting PGH1801, *S. aureus* Newman could produce a unique phenotypic adaptation. This property is known as phenotypic plasticity [16]. Such ‘transgenerational’ plasticity could provide a competitive advantage in growth resulting in long-term environmental variation [17,18,19,20].

Next-generation sequencing allows the study of any organism at the genomic and transcriptomic levels. Exploring whole-genome expression using next-generation sequencing and RNA-seq provides a more comprehensive understanding than just looking at DNA primary structure, since RNA-seq captures actively transcribed regions and, therefore, can ascertain the molecular basis of a phenotype [21,22,23,24,25]. In this study, we have applied DNA- and RNA-seq analyses by comparing genome-wide single nucleotide polymorphism (SNP) and insertion/deletion (*InDel*) results, as well as by measuring the differential expression levels of thousands of genes simultaneously between the strains of wild-type Newman_2010 and mutant 1801_2010 to identify mutations associated with the above resistant phenotypes. Meanwhile, our comparative transcriptome analysis and *de novo* short-read assembly revealed the transcriptional changes of *S. aureus* in response to lysostaphin treatment. All the pooled RNA-seq data were used to compare and establish genome-wide SNP and *InDel* results between the mutant 1801_2010 and WT Newman_2010. Reverse transcription qPCR was used to confirm the RNA-seq results. Computational analyses, including gene ontology and KEGG pathway enrichment, were also employed. We aim to identify the mutations which provide resistance to the triple-acting PGH. The findings in this work could provide insights into the design of new antimicrobial agents.

## 2. Materials and Methods

### 2.1. Bacterial Strains, Culture Conditions and Mutant Isolation

*S. aureus* strain Newman_2010 (wild-type) was used in this study [2]. A mutant strain 1801_2010 was developed by repeated exposure of the wild-type strain to sublethal concentrations of the triple-acting PGH1801, a fusion protein containing 3 active domains [2,12,14]. Briefly, *S. aureus* Newman 2010 bacteria were incubated with two-fold serial dilutions of PGH1801 (10, 5, 2.5, 1.25, and 0.63 µg/well). On day 1 of the MIC assay, bacteria growing in the first turbid well (a sublethal concentration) next to clear wells (lethal concentration) were selected as an inoculate for next MIC assay. Over this time of repeated exposures, bacteria developed more and more resistance to PGH1801. After 10 cycles of exposure of *S. aureus* Newman_2010 to sublethal concentrations of PGH1801, a mutant strain (1801_2010) with a 2- fold increase in MIC was isolated. Both wild-type and mutant *S. aureus* strains were grown at 37 °C on tryptic soy agar (TSA) or in tryptic soy broth (TSB). Growth curves of the strains were determined and shown in Supplementary Figure S1. The wild-type and mutant strains displayed a similar growth pattern with the doubling times 47.9 ± 0.431 and 48.1 ± 0.458 min, respectively.

### 2.2. RNA Preparation

*S. aureus* Newman_2010 and 1801_2010 cultures were grown in triplicates to around 0.4 of OD600, and harvested by centrifugation at 4000× *g* for 15 min at 4 °C. Bacterial pellets were then immediately resuspended into TRI Reagent (Molecular Research Center, Inc., Cincinnati, OH, USA). Total RNA was extracted using Direct-Zol RNA MiniPrep according to the manufacturer’s instructions (Zymo Research, Irvine, CA, USA). Each RNA sample (50 μg) was treated with 5 U of RNase-free DNase I in the presence of 80 U of RNasin (Promega) at 37 °C for 2 h to remove traces of chromosomal DNA, and then purified using an RNA Clean and Concentrator™-25 kit (Zymo Research, Irvine, CA, USA). Eluted RNAs were quantified using a Qubit 2.0 fluorometer with a Qubit RNA HS Assay Kit (Life Technologies, Carlsbad, CA, USA). Quality of each RNA sample was evaluated using an Agilent Bioanalyzer (Agilent Technologies, Santa Clara, CA, USA) with an RNA 6000 Nano kit. All the samples showed the RNA integrity numbers (RIN) above 8. The efficiency of DNase I treatment of the RNA samples was assessed by PCR amplification of a *S. aureus* house-keeping gene (*gyrA*) with a positive (*S. aureus* genomic DNA) and negative control (nuclease-free water).

### 2.3. RNA-seq Library Construction and Sequencing

Prior to RNA-seq library preparation, ribosomal RNA was depleted from the total RNA using a Ribo-Zero rRNA Removal Kit for Gram-positive Bacteria (Illumina, San Diego, CA, USA) according to the manufacturer’s instructions. A Qubit RNA HS Assay and Agilent Bioanalyzer with an RNA 6000 Pico kit were used to assess the quantity and quality of the rRNA-depleted RNA samples. Six libraries (2 strains × 3 biological replicates) were constructed using a TruSeq Stranded mRNA Sample Preparation kit (Illumina, San Diego, CA, USA) following the manufacturer’s recommendations. The libraries were sequenced on an Illumina Miseq platform using 300 base- length read chemistry in a paired-end mode.

### 2.4. Reverse Transcription Quantitative PCR (RT-qPCR) Analysis

RT-qPCR was performed on selected genes based on the relative transcription levels (logFC > 2.0) obtained from RNA-seq results. Primers were designed using the NCBI primer designing tool (http://www.ncbi.nlm.nih.gov/tools/primer-blast/index.cgi) (accessed on 10 January 2022). Reverse transcription reactions were carried out using random primers and SuperScript II Reverse Transcriptase (Life Technologies, Carlsbad, CA, USA). Quantification of cDNA was performed on a 7500 real-time PCR system (Applied Biosystems, Foster City, CA, USA) using a SYBR^®®^ Green PCR Master Mix. The *gyrA* gene was used as a reference for data normalization. Housekeeping genes *gmk* and *pta* were included as controls to ensure data reliability. All the samples were analyzed in three biological and technique triplicates. Relative gene expression levels were computed by the 2^−ΔΔCT^ method, where ΔΔCT = ΔCT (mutant) − ΔCT (WT), ΔCT = CT (target gene) − CT (*gyrA*), and CT is the threshold cycle value of the amplified gene.

### 2.5. Bioinformatics Analysis

Computational analyses, including gene ontology and KEGG pathway enrichment analysis, were performed [26]. Pooled RNA-seq data were used to compare genome-wide SNP and *InDel* results between the mutant 1801_2010 and WT Newman_2010 strains. The details are referenced [27].

### 2.6. Data Accession Numbers

Project accessions in NCBI: PRJNA235858 and PRJNA235865 for *S. aureus* newman_2010 and 1801_2010 strains are available, respectively. DNA sequence accession numbers are SRX478042 (Newman_2010) and SRX478056 (1801_2010). The whole-genome sequence of *S. aureus* Newman_WT (GenBank accession: NC_009641) was used as a reference for genome mapping.

## 3. Results

### 3.1. Integrated Genome and Transcriptome Sequencing for Identification of Genetic Variants

**Figure 1:**
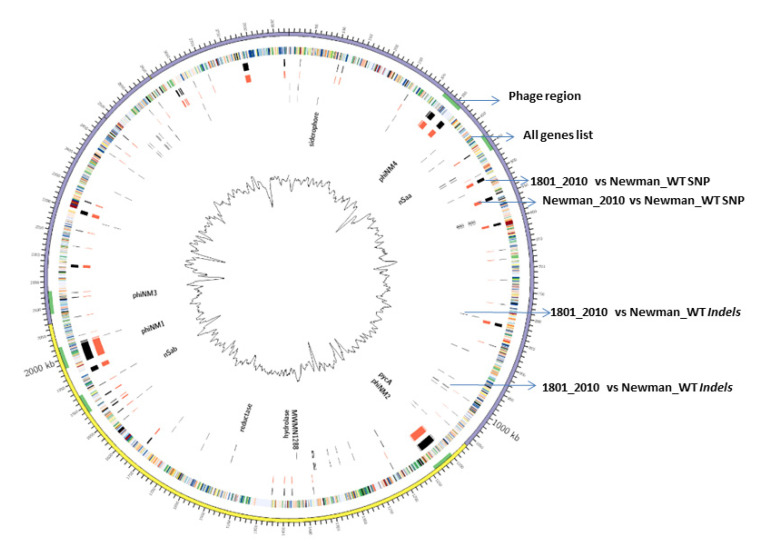
WGS and RNA-seq polymorphism comparison. Ring 1 (outer circle) shows the chromosomal map positions; ring 2 lists chromosome genes; rings 3 and 4 represent the SNP differences between *S. aureus* mutant 1801_2010 to Newman_WT and Newman_2010 to Newman_WT; rings 5 and 6 represent the *InDel* differences between *S. aureus* mutant 1801_2010 to Newman_WT and Newman_2010 to Newman_WT.

In this study, we applied a Circos diagram (Figure 1) to illuminate the whole-genome and transcriptome sequencing in order to graphically represent genetic variations and association with their corresponding phenotypes across *S. aureus* Newman WT and mutant strains. Figure 1 shows several genetic differences between mutant 1801_2010 and WT Newman_2010 compared to the reference genome of Newman_WT (NC_009641).

### 3.2. InDels, Gaps, and SNPs Identified

**Figure 2:**
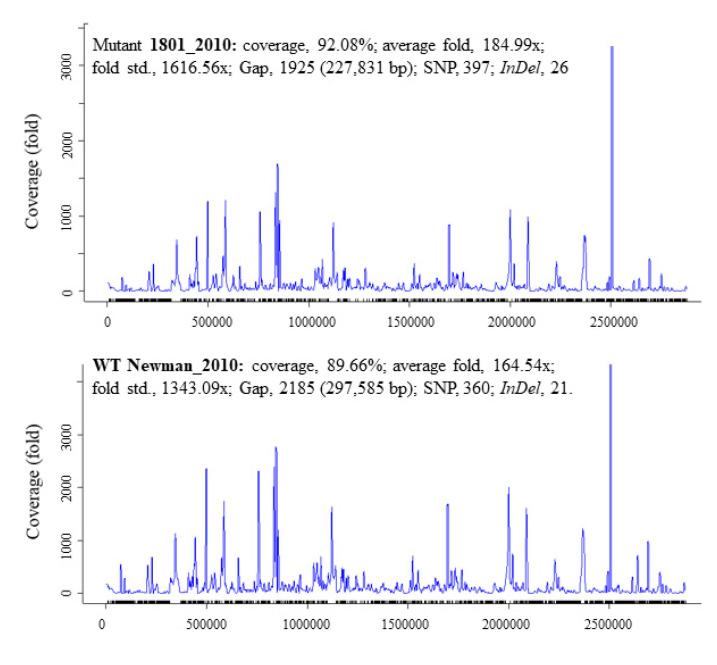
Summary statistics for the *InDel* and SNP data of *S. aureus* Newman_2010 and mutant 1801_2010 by comparing to Newman_WT (NC_009641).

There was a total of 397 SNPs, 1925 gaps, and 26 *InDels* detected in mutant 1801_2010 and 360 SNPs, 2185 gaps, and 21 *InDels* in WT Newman_2010 (Figure 2) compared to Newman_WT *via* using the EDGE bioinformatics software [27]. RNA-seq data were used to verify these results. The purpose of this study is to investigate whether these genomic alterations contribute to gene expression. There are six rings in Figure 1: the inner two rings are the genetic difference of *InDels*, the middle two rings are SNP differences. The second ring from the outside is the list of all genes. All genetic variations of SNPs and *InDels* from WT Newman_2010 and mutant 1801_2010 were mapped relative to the reference genome sequence of Newman_WT (NC_009641). Several important genes have been identified which could be responsible for the phenotype. Table 1 lists the polymorphic changes in the mutant compared to Newman_WT. Of the 21 significant SNPs, 18 were associated with 4 individual genes (NWMN_0305, NWMN_0306, NWMN_0979, and NWMN_1288). The remaining three SNPs were distributed in the intergenic regions: two of them were between the *lctp* and *Spa* genes and one was located between the *recR* and *tmk* genes. Table 2 presents the 15 *InDels* identified in mutant 1801_2010 compared to Newman_WT. Two genes (NWMN_1308 and NWMN_1410) contained 5 *InDels*. The remaining 10 *InDels* were distributed in 5 important intergenic regions: 4 of them were in the intergenic region between the NWMN_0810 and NWMN_0811 genes; 2 were in the intergenic region between the NWMN_2500 and *ldh* genes; 2 of the *InDels* were in the intergenic region between the *citZ* and *aapA* genes; 2 of them were in the region between *dnaA* and *rpmH*. In Table 1 and Table 2, two different assembly methods were compared to identify genetic variations: one was based on sequence reads and the other was based on contigs. The results from both methods agreed with each other very well.

### 3.3. Identification of Up-Regulated and Down-Regulated Genes

After normalization, the DESeq2 tool [28] was employed for quantitative analysis of RNA-seq data and identification of the genes differentially expressed between the mutant and WT strains. In total, 1091 differentially expressed genes (DEGs) (padj < 0.001) were obtained (Supplementary Table S1): 18 of them were significantly up-regulated (padj < 0.001, log2 FC > 0.7, FDR < 0.05) and 6 genes were significantly down-regulated (padj < 0.001, log2 FC < −0.7, FDR < 0.05). The summary of DEGs between the mutant 1801_2010 and WT Newman_2010 is shown in Table 3. Comparative transcriptome analysis and *de novo* short-read transcriptome assembly revealed that significant transcriptional changes in response to the triple-acting PGH 1801 were associated with membrane, cell wall, and their related genes (e.g., amidase, peptidase, holin, and phospholipase D/transphosphatidylase).

### 3.4. Function Ontology and KEGG Pathway Enrichment Analyses of DEGs

**Figure 3:**
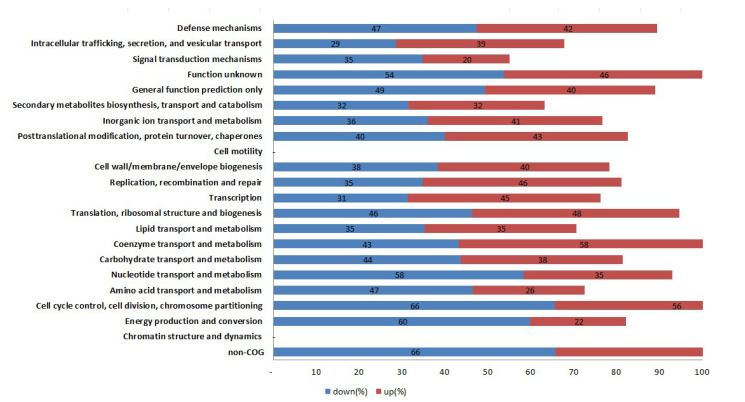
KEGG pathway classification of the DEGs between *S. aureus* mutant 1801_2010 and WT Newman_2010.

To annotate the potential functions of the DEGs between WT and mutant strains, DEGs with >2-fold expression change were assigned to different KEGG pathways. All KEGG pathways were analyzed as shown in Figure 3. In these pathways, energy production and conversion, amino acid transport and metabolism, nucleotide transport and metabolism, intracellular trafficking, secretion, and vesicular transport, signal transduction and mechanisms were the most enriched pathways either up- or down-expressed between WT Newman_2010 and mutant 1801_2010 strains.

### 3.5. RT-qPCR Confirmation

**Figure 4:**
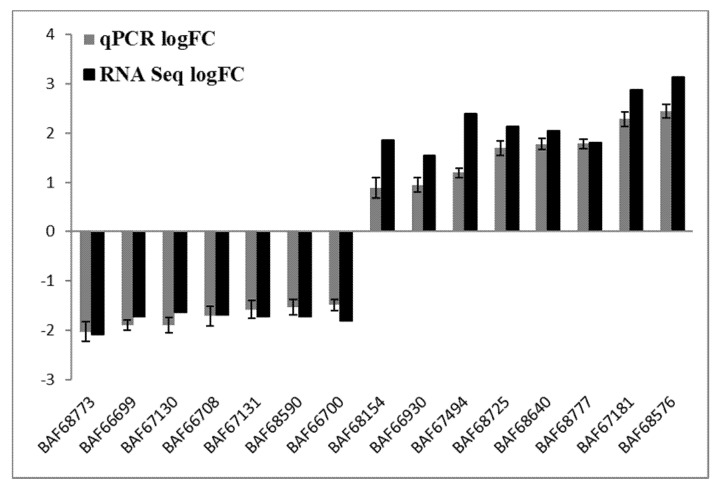

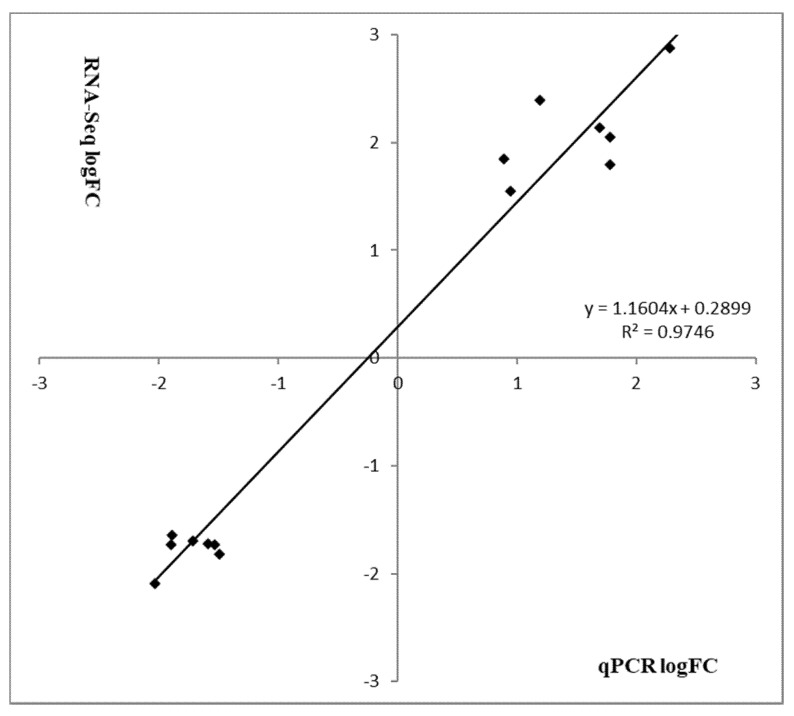
Validation of RNA-seq analysis by RT-qPCR. Relative gene expression levels of *S. aureus* mutant 1801_2010 and WT Newman_2010 were quantified by RT-qPCR, and data were analyzed using the comparative critical threshold (ΔΔCT) method. The ratios (log2) of relative gene expression from RT-qPCR and RNA-seq are shown in grey and black bars, respectively. A ratio greater than zero (>0) indicates up regulation of gene expression and a ratio below zero (<0) indicates down regulation in mutant 1801_2010. Error bars indicate standard deviations of three replicates.

To verify the most differentially expressed genes obtained from RNA-seq datasets of WT Newman_2010 and mutant 1801_2010, we performed reverse transcription qPCR experiments and compared these results with RNA-seq. Figure 4 demonstrated that the changes of gene expression from RT-qPCR correlated well with the transcriptome profiling from RNA-seq. Moreover, these results were consistent with the observed structural changes at the DNA and RNA levels (Table 1 and Table 2). These structural changes in the genes encoding membrane/cell surface proteins and the perturbation in gene expression are potential candidates responsible for resistance to these novel antimicrobials.

## 4. Discussion

Our previous genome sequencing work (https://www.ncbi.nlm.nih.gov/sra/?term=SRX478056) (accessed on 10 January 2022) has been trying to identify the genomic changes in *Staphylococcus aureus* that confer resistance to peptidoglycan hydrolase antimicrobial enzymes [15,29]. In this study, we combined RNA-seq and genome sequence data of WT Newman_2010 and mutant 1801_2010 strains by comparing genome-wide SNP and *InDel* results. Additionally, we applied RT-qPCR to confirm these results. To reveal the differences between the positive genes and other genes, computational analyses, including gene ontology and KEGG pathway enrichment, were also employed. These differences between mutant 1801_2010, WT Newman_2010, and Newman_WT strains in Figure 1 indicate these genetic changes are potentially responsible for phenotypic variation. However, a phenotypic trait caused by genetic sources of variation could include additive variance, dominant variance, environmental variance (e.g., organismal adaptation), and epistatic variance [30,31].

In Table 1 and Table 2, and Figure 2, we observed the genetic variations which could be responsible for the mechanism of resistance to peptidoglycan hydrolase (PGH 1801) in *S. aureus.* However, these variations are only statistical indicators of a functional effect associated with their genotypic variants because it is uncommon to have a concrete variant with a “precise” genetic location with measurable statistical “effects”. Although we can say that there is a functional effect, in order to determine functionality we must go beyond the identification of the variant phenotype due to a specific SNP- or *InDel*-based locus. It will be instructive to compare the results of current study with those in the literatures. Mostly, there are genomic loci that could influence the expression level of mRNA and these loci can be physically located close to or far away from the gene that gets regulated. It is not necessary that genetic loci are associated with a SNP or *InDel*.

In this study, we observed significant transcriptional changes in *S. aureus* Newman_2010 upon exposure to the triple-acting fusion protein PGH1801 [2,12]. We found most of these are membrane proteins, cell wall related proteins [32,33,34,35,36,37], such as amidase [12], peptidase [29], holin [38,39,40,41,42], and phospholipase D/transphosphatidylase [43,44].

Through the Gene Ontology function and KEGG pathway enrichment analyses, we found energy production and conversion, amino acid transport and metabolism, nucleotide transport and metabolism, intracellular trafficking, secretion, and vesicular transport, signal transduction and mechanisms were the most enriched pathways either up- or down-expressed between the wild-type and mutant.

Comparative transcriptome analysis and *de novo* short-read assembly in this study revealed that the genes with significant transcriptional changes in response to exposure to the triple-acting fusion protein are associated with membrane and cell wall (e.g., amidase, peptidase, holin, and phospholipase D/transphosphatidylase). These results are consistent with the observed nucleotide changes at the DNA level. The nucleotide changes in the genes encoding membrane/cell surface proteins and the alteration of gene expression may contribute to the increased resistance of *S. aureus* to PGHs. The findings of this study could provide insights into the target genes responsible for PGH resistance and lead to the design of new antimicrobial agents.

## Figures and Tables

**Table 1:** Polymorphic changes in *S. aureus* mutant 1801_2010 compared to Newman_WT *.

Technology	Sample	Method	SNP_position	Ref_codon	Sub_codon	Ref_aa	Sub_aa	Synonymous	Product	CDS_start	CDS_end
WGS	mutant	contigs	73,365	C	T				Intergenic region (between *lctP* and *spa*)		
WGS	mutant	reads	73,365	C	T				Intergenic region (between *lctP* and *spa*)		
WGS	mutant	contigs	354,546	GCG	GCA	A	A	Yes	NWMN_0305: hypothetical protein	353,131	355,029
WGS	mutant	contigs	354,585	GGT	GGG	G	G	Yes	NWMN_0305: hypothetical protein	353,131	355,029
WGS	mutant	contigs	354,594	TTT	TTG	F	L	No	NWMN_0305: hypothetical protein	353,131	355,029
WGS	mutant	contigs	354,729	TGT	TGC	C	C	Yes	NWMN_0305: hypothetical protein	353,131	355,029
WGS	mutant	contigs	354,767	AAT	AGT	N	S	No	NWMN_0305: hypothetical protein	353,131	355,029
WGS	mutant	contigs	354,859	TTT	CTT	F	L	No	NWMN_0305: hypothetical protein	353,131	355,029
WGS	mutant	contigs	354,906	CCG	CCA	P	P	Yes	NWMN_0305: hypothetical protein	353,131	355,029
WGS	mutant	contigs	355,079	ACA	ACG	T	T	Yes	NWMN_0306: hypothetical protein	355,029	356,852
WGS	mutant	contigs	355,208	AAG	AAA	K	K	Yes	NWMN_0306: hypothetical protein	355,029	356,852
WGS	mutant	contigs	355,674	GTC	TTC	V	F	No	NWMN_0306: hypothetical protein	355,029	356,852
WGS	mutant	contigs	504,664	T	C				Intergenic region (between *recR* and *tmk*)		
WGS	mutant	contigs	1,086,625	AGC	AGT	S	S	Yes	NWMN_0979 (*pycA*): pyruvate carboxylase	1,085,501	1,088,971
WGS	mutant	reads	1,086,625	AGC	AGT	S	S	Yes	NWMN_0979 (*pycA*): pyruvate carboxylase	1,085,501	1,088,971
RNA-seq	mutant	contig	1,086,625	AGC	AGT	S	S	Yes	NWMN_0979 (pycA): pyruvate carboxylase	1,085,501	1,088,971
RNA-seq	mutant	reads	1,086,625	AGC	AGT	S	S	Yes	NWMN_0979 (*pycA*): pyruvate carboxylase	1,085,501	1,088,971
WGS	mutant	contigs	1,420,272	ATT	GTT	I	V	No	NWMN_1288: hypothetical protein	1,420,254	1,421,021
WGS	mutant	reads	1,420,272	ATT	GTT	I	V	No	NWMN_1288: hypothetical protein	1,420,254	1,421,021
RNA-seq	mutant	contig	1,420,272	ATT	GTT	I	V	No	NWMN_1288: hypothetical protein	1,420,254	1,421,021
RNA-seq	mutant	reads	1,420,272	ATT	GTT	I	V	No	NWMN_1288: hypothetical protein	1,420,254	1,421,021

* A wild-type Newman strain (NC_009641) was used as a reference in the data analysis.

**Table 2:** *InDels* identified in *S. aureus* mutant 1801_2010 compared to Newman_WT *.

Technology	Sample	Method	InDel_position	Sequence	InDel_seq	Length	Type	Product	CDS_start	CDS_end
DNAseq	WT	reads	2	GAT	GATCGAT	4	Insertion	Intergenic region (between *dnaA* and *rpmH*)		
DNAseq	mutant	reads	4	TT	TTTTTATCGATT	10	Insertion	Intergenic region (between *dnaA* and *rpmH*)		
RNA-seq	mutant	reads	897,844	GC	GCC	1	Insertion	Intergenic region (between NWMN_0810 and NWMN_0811)		
DNAseq	mutant	reads	897,844	GC	GCC	1	Insertion	Intergenic region (between NWMN_0810 and NWMN_0811)		
DNAseq	mutant	reads	897,951	TG	TGG	1	Insertion	Intergenic region (between NWMN_0810 and NWMN_0811)		
RNA-seq	mutant	reads	897,989	AT	ATT	1	Insertion	Intergenic region (between NWMN_0810 and NWMN_0811)		
DNAseq	mutant	reads	1,441,311	ACCC	ACC	1	Deletion	NWMN_1308 (*dapD*) :tetrahydrodipicolinate acetyltransferase	1,440,676	1,441,395
DNAseq	mutant	reads	1,441,331	CA	CAA	1	Insertion	NWMN_1308 (*dapD*) :tetrahydrodipicolinate acetyltransferase	1,440,676	1,441,395
DNAseq	mutant	reads	1,578,835	GAAA	GAA	1	Deletion	NWMN_1410:pyrroline-5- carboxylate reductase	1,578,193	1,579,008
RNA-seq	mutant	reads	1,578,900	GCC	GC	1	Deletion	NWMN_1410:pyrroline-5- carboxylate reductase	1,578,193	1,579,008
DNAseq	mutant	reads	1,578,900	GCC	GC	1	Deletion	NWMN_1410:pyrroline-5- carboxylate reductase	1,578,193	1,579,008
DNAseq	mutant	reads	1,761,003	ATTTTTT	ATTTTT	1	Deletion	Intergenic region (between *citZ* and *aapA*)		
DNAseq	mutant	contigs	1,761,008	T		1	Deletion	Intergenic region (between *citZ* and *aapA*)		
RNA-seq	mutant	contigs	2,744,980	G		1	Deletion	Intergenic region (between NWMN_2500 and *Ldh*)		
DNAseq	mutant	contigs	2,744,980	G		1	Deletion	Intergenic region (between NWMN_2500 and *Ldh*)		
DNAseq	mutant	reads	2,878,891	CTTTTA T	CTTTTATCGATTT TAT	9	Insertion	Intergenic region (between *dnaA* and *rpmH*)		

* A wild-type Newman strain (NC_009641) was used as a reference in the data analysis.

**Table 3:** Up- and down-regulated genes in *S. aureus* mutant 1801_2010 compared to WT Newman_2010.

Down-regulated gene (higher expression in WT)						
Gene_symbol	Gene_ID	LogFC	LogCPM	p-Value	FDR	Protein name
NWMN_0078	BAF66350	−0.83	7.32	0.00	0.01	surface protein SasD
*gntR*	BAF66470	−0.92	5.12	0.00	0.03	GntR
NWMN_0738	BA67010	−0.71	8.16	0.00	0.04	Conserved hypothetical protein
NWMN_1951	BAF68223	−1.08	4.81	0.00	0.04	oxidoreductase
*lukF*	BAF68199	−0.95	6.15	0.00	0.04	gamma-hemolysin subunit B
NWMN_2209	BAF68481	−0.83	6.91	0.00	0.04	conserved hypothetical protein
Up-regulated gene (lower expression in WT)						
NWMN_2304	BAF68576	3.13	4.99	0.00	0.00	membrane protein
NWMN_1882	BAF68154	1.85	4.17	0.00	0.00	holin (holin, toxin secretion/phage lysis family protein)
NWMN_0537	BAF66809	1.05	11.68	0.00	0.00	membrane protein
NWMN_1068	BAF67340	1.21	4.88	0.00	0.00	conserved hypothetical protein
NWMN_0909	BAF67181	2.88	2.60	0.00	0.00	membrane protein
NWMN_2505	BAF68777	1.69	4.27	0.00	0.00	membrane protein
NWMN_1874	BAF68146	1.18	5.74	0.00	0.01	putative membrane protein
NWMN_2287	BAF68559	0.69	7.66	0.00	0.01	hsp20-like protein
NWMN_1639	BAF67911	0.77	6.91	0.00	0.01	peptidase
NWMN_1256	BAF67528	0.72	6.40	0.00	0.03	cytochrome C biogenesis protein *CcdC*
NWMN_0985	BAF67257	0.91	5.07	0.00	0.03	conserved hypothetical protein
*nrdH*	BAF67223	1.24	4.55	0.00	0.04	NrdH-redoxin
NWMN_2223	BAF68495	0.73	6.62	0.00	0.04	conserved hypothetical protein
NWMN_1881	BAF68153	0.78	6.25	0.00	0.04	amidase
NWMN_2154	BAF68426	0.83	6.67	0.00	0.04	probable membrane protein
NWMN_0920	BAF67192	0.99	4.49	0.00	0.04	acyltransferase
NWMN_0986	BAF67258	0.81	5.86	0.00	0.05	conserved hypothetical protein
NWMN_1229	BAF67501	1.43	3.40	0.00	0.05	phospholipase D/transphosphatidylase

## Data Availability

DNA sequence reads are available in GenBank of NCBI with the accession No. SRX478042 for *S. aureus* Newman_2010 and SRX478056 for mutant 1801_2010. The GenBank accession number for complete genome sequence of *S. aureus* Newman_WT is NC_009641.

## Supplementary Materials

The following supporting information can be downloaded at: https://www.mdpi.com/article/10.3390/antibiotics11020125/s1, Figure S1: Growth curves of *S. aureus* Newman_2010 and mutant 1801_2010; Table S1: Complete list of differentially expressed genes based on normalized RNA-seq data.
