# Supplementary material for: Comparative Transcriptome Analysis Reveals Differentially Expressed Genes Related to Antimicrobial Properties of Lysostaphin in Staphylococcus aureus
Source: Antibiotics (Basel). 2022 Jan 18;11(2):125. doi: 10.3390/antibiotics11020125 (PMC8868216; doi:10.3390/antibiotics11020125)
Supplement: Supplementary file 1 [file antibiotics-11-00125-s001.zip › antibiotics-1530762-figure s1.pdf]

antibiotics

MDPI

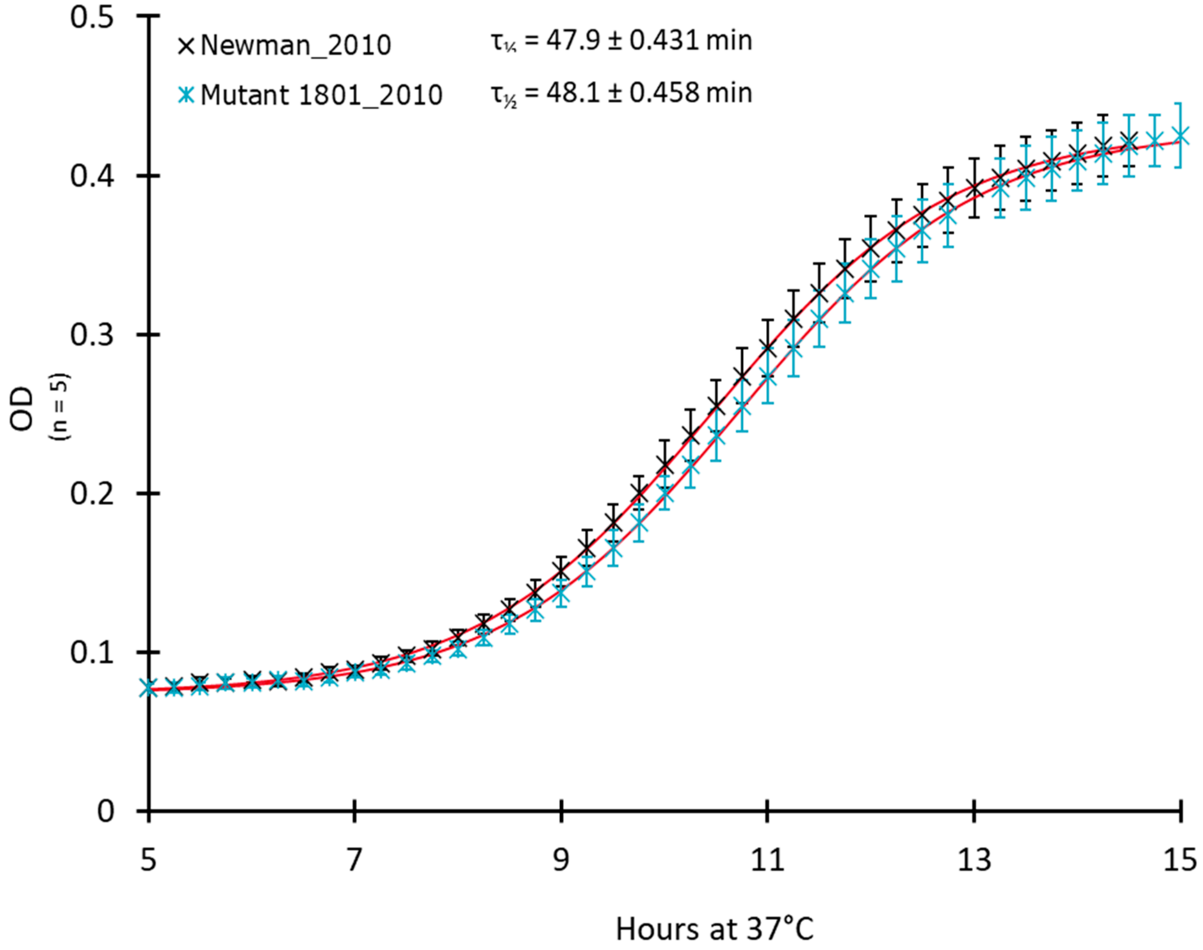

Figure S1. Growth curves of *S. aureus* Newman_2010 and mutant 1801_2010.Five replicates of bacterial cultures grown at 37ºC in tryptic soy broth (TSB) were used for the calculation of growth curves and standard deviations. The doubling time ($\tau_{½}$) for each strain is indicated in the chart.
